# Cancer‐related thrombotic microangiopathy and disseminated intravascular coagulation in a patient with bone marrow carcinomatosis of unknown primary origin: A case report

**DOI:** 10.1002/cnr2.2036

**Published:** 2024-03-22

**Authors:** Masahiro Manabe, Naoyuki Inano, Yuuji Hagiwara, Nobuhiro Sogabe, Satoru Nanno, Takeshi Mazaki, Ki‐Ryang Koh

**Affiliations:** ^1^ Department of Hematology Osaka General Hospital of West Japan Railway Company Osaka Japan; ^2^ Department of Clinical Laboratory Osaka General Hospital of West Japan Railway Company Osaka Japan; ^3^ Department of Pathology Osaka General Hospital of West Japan Railway Company Osaka Japan

**Keywords:** bone marrow carcinomatosis, Cancer‐related thrombotic microangiopathy, carcinoma of unknown primary, PLASMIC score

## Abstract

**Background:**

Cancer‐related thrombotic microangiopathy (CR‐TMA) is a rare type of Coombs‐negative hemolytic anemia, which is caused by malignancy and has a poor prognosis.

**Case:**

A 76‐year‐old female was referred to our hospital due to Coombs‐negative hemolytic anemia, which was causing fatigue and dyspnea on exertion, accompanied by schistocytosis. A bone marrow examination demonstrated bone marrow carcinomatosis, and the tumor cells were morphologically suspected to be signet‐ring cell carcinoma cells. As we failed to find the primary tumor site before the patient died, she was diagnosed with CR‐TMA due to bone marrow carcinomatosis of unknown primary origin. Thrombotic thrombocytopenic purpura (TTP) was rapidly ruled out based on her PLASMIC score. In addition, immunohistochemical staining of a clot section of the bone marrow and tumor marker data were useful for narrowing down the likely primary tumor site.

**Conclusion:**

Although CR‐TMA is an extremely rare phenomenon, clinicians who suspect CR‐TMA should quickly rule out TTP and decide whether to provide appropriate chemotherapy or plan for palliative care.

## 1 INTRODUCTION

Cancer‐related thrombotic microangiopathy (CR‐TMA) is a rare paraneoplastic syndrome, which is most commonly seen in gastric cancer, followed by breast, prostate, and lung cancer.^1^ It is usually associated with advanced disease and has a poor prognosis.^1^, ^2^, ^3^ In this report, we describe the case of a 76‐year‐old patient, who presented with CR‐TMA due to bone marrow carcinomatosis of unknown primary origin. Although the primary lesion could not be identified before the patient died, immunostaining of bone marrow specimens was useful for narrowing down its origin.

## 2 CASE PRESENTATION

**FIGURE 1:**
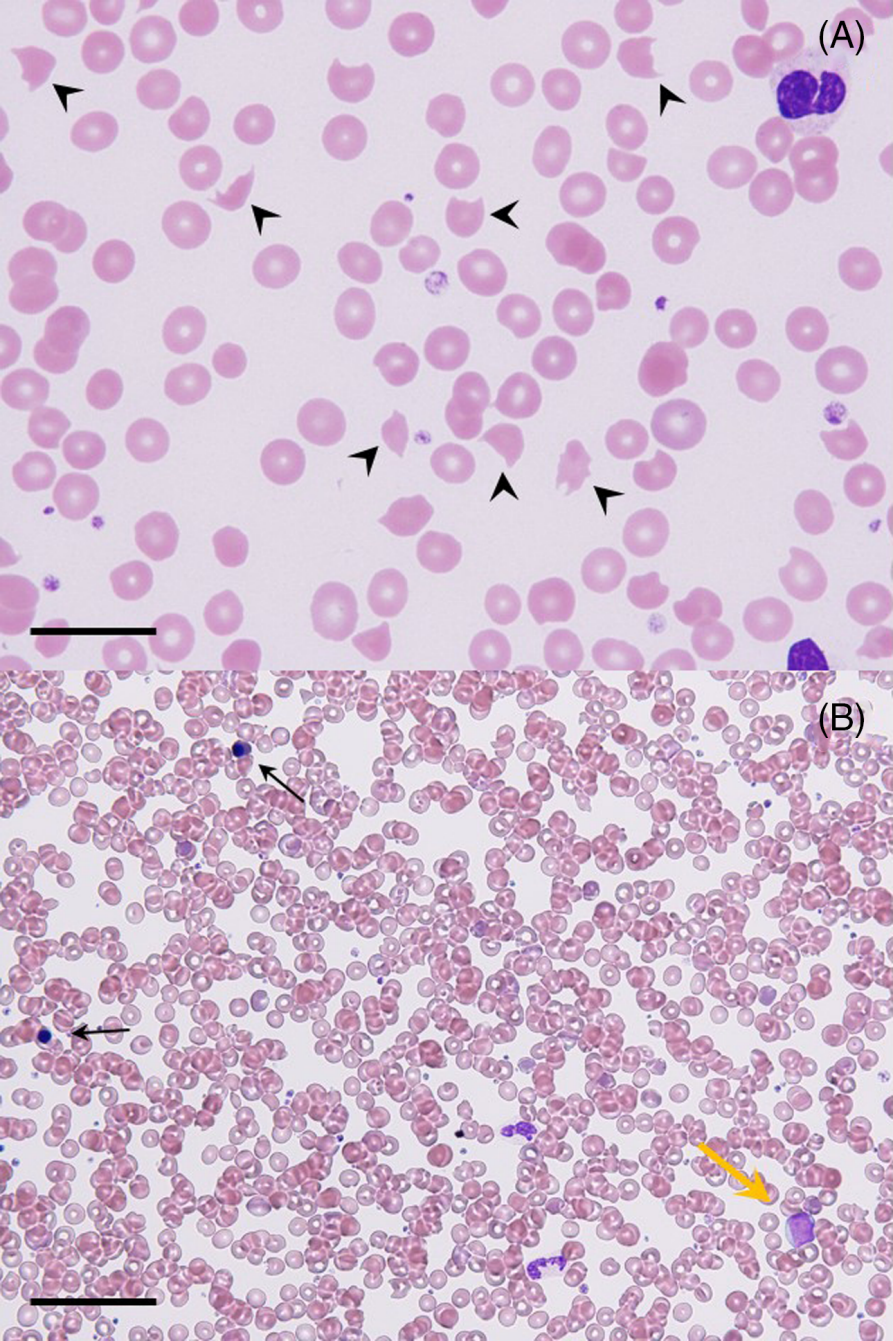
Peripheral blood smear obtained at diagnosis (May‐Giemsa staining). (A) Prominent schistocytes were seen (arrowheads, original magnification: ×1000, scale bar: 20 μm). (B) Nucleated red cells (black arrows) and immature myeloid precursors (yellow arrow) were seen (original magnification: ×200, scale bar: 100 μm).

**FIGURE 2:**
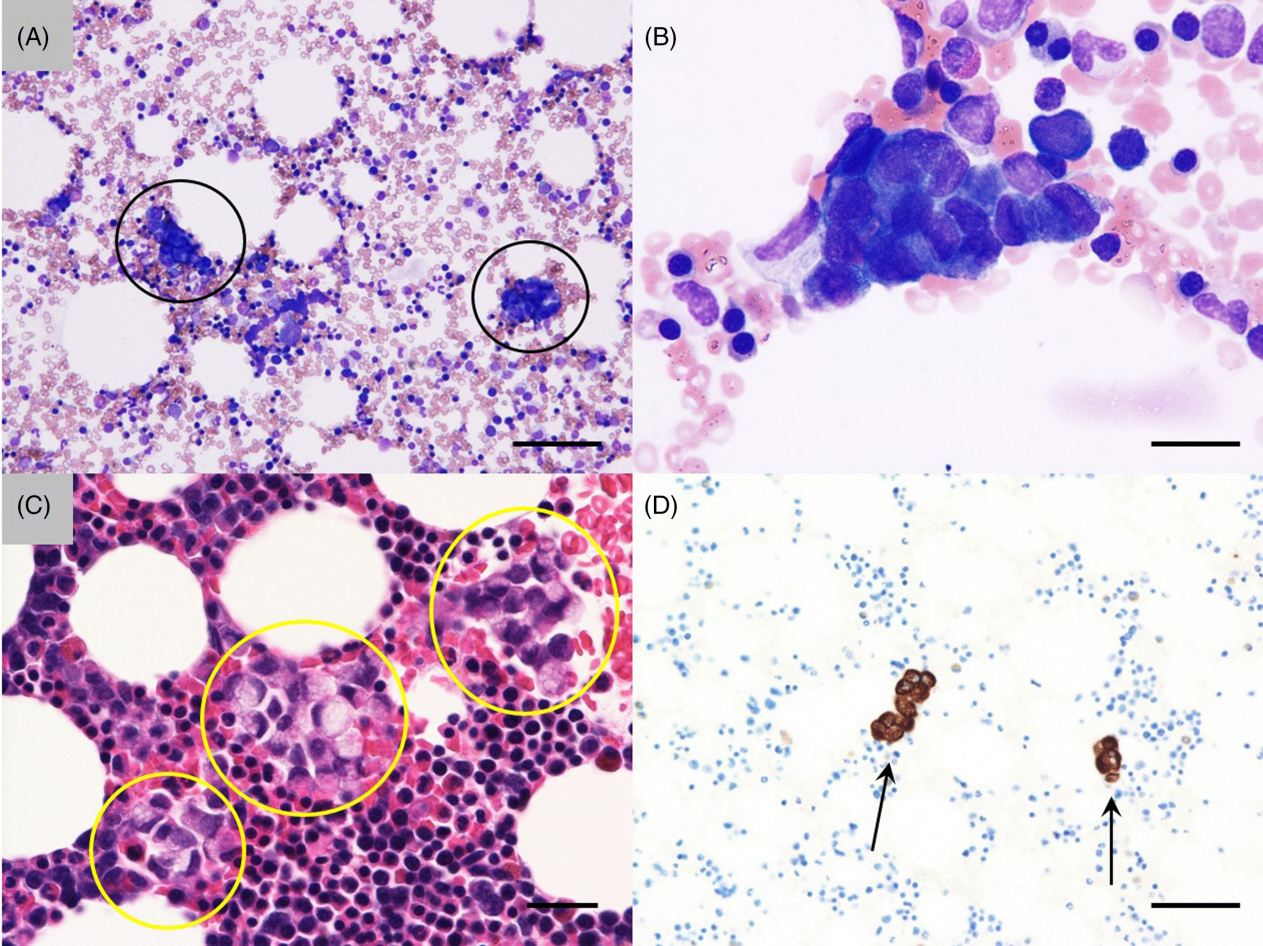
Bone marrow examination. (A) A bone marrow smear accompanied by bone marrow carcinomatosis (black circles, May‐Giemsa staining, original magnification: ×200, scale bar: 100 μm); (B) A cluster of carcinoma cells (May‐Giemsa staining, original magnification: ×1000, scale bar: 20 μm); (C, D) Pathological images of a bone marrow clot section Carcinoma cells harbor eccentrically located nuclei, which were compatible with signet‐ring cell carcinoma, were seen (C, yellow circles, hematoxylin–eosin staining, original magnification: ×400, scale bar: 40 μm), and immunohistochemistry demonstrated that these cells were positive for CK20 (D, arrows, original magnification: ×200, scale bar: 100 μm).

A 76‐year‐old female was referred to our hospital (Osaka General Hospital of West Japan Railway Company, Osaka, Japan) due to a weeklong history of fatigue and dyspnea on exertion. A physical examination revealed pallor and icterus. The surface lymph nodes, liver, and spleen were not palpable. She had no relevant medical history and was not taking any medication. Although her brother had experienced gastric carcinoma, there was no family history of hemolysis. She was found to have hemolytic anemia (hemoglobin concentration, 9.2 g/dL [reference range, 12.0–16.0 g/dL]; mean corpuscular volume (MCV), 84.5 fL [reference range, 89–99 fL]; platelets, 153 × 10^9^/L [reference range, 130–320 × 10^9^/L]; white blood cells (WBC), 7.4 × 10^9^/L [reference range, 3.4–8.4 × 10^9^/L]; lactate dehydrogenase (LDH), 1823 U/L [reference range, 125–220 U/L]; total bilirubin, 8.8 mg/dL [reference range, 0.2–1.2 mg/dL]; direct bilirubin, 0.7 mg/dL [reference range, 0–0.5 mg/dL]; and haptoglobin, <10 mg/dL [reference range: type 1‐1, 83–209 mg/dL; type 2‐1, 66–218 mg/dL; type 2‐2, 25–176 mg/dL]). Direct and indirect Coombs tests were negative. A peripheral blood film revealed marked schistocytosis (Figure 1), and erythroblastosis was also seen (4/100WBC). In addition, disseminated intravascular coagulation (DIC) was also noted (prothrombin time (PT), 13.2 s [reference range, 9.5–11.8 s]; activated partial thromboplastin time (APTT), 28.8 s [reference range, 26.9–38.1 s]; fibrinogen, 120 mg/dL [reference range, 200–400 mg/dL]; fibrin degradation products (FDP), 103.3 μg/mL [reference range, 0–5 μg/mL]; and d‐dimer, 15.8 μg/mL [reference range, 0–1 μg/mL]). Although thrombotic thrombocytopenic purpura (TTP) was considered as a differential diagnosis, the patient's ADAMTS13 (a disintegrin and metalloproteinase with a thrombospondin type 1 motif, member 13) activity was normal (95% [reference range, >10%]), according to an enzyme‐linked immunosorbent assay. A bone marrow smear showed a normocellular bone marrow with clusters of carcinoma cells, and a bone marrow clot specimen demonstrated the metastasis of carcinoma cells with unevenly distributed nuclei, suggesting signet ring‐cell carcinoma (Figure 2). Although immunohistochemical staining of bone marrow clot sections showed positivity for cytokeratin (CK) 20, staining for CK7, the estrogen receptor, GATA‐binding protein 3 (GATA3), and caudal type homeobox transcription factor 2 (CDX2) were negative. Although immunohistochemical staining to test for mismatch repair (MMR) deficiency (mutL homolog 1 [MLH1], PMS1 homolog 2 [PMS2], mutS homolog 2 [MSH2], and mutS homolog 6 [MSH6] staining) was also performed, the results were inconclusive since there were few clusters of cancer cells in the bone marrow. The patient's tumor marker levels were as follows: carcinoembryonic antigen, 17759.9 ng/mL (reference range, 0–5 ng/mL); carbohydrate antigen 19–9, <2.0 U/mL (reference range, 0–37 U/mL); alpha‐fetoprotein, 3.5 ng/mL (reference range, 0–13.4 ng/mL); and neuron‐specific enolase, 75.2 ng/mL (reference range, 0–16.3 ng/mL). Upper gastrointestinal endoscopy and whole‐body computed tomography (CT) revealed no abnormalities that were suggestive of primary carcinoma, and CT demonstrated no evidence of infectious disease. Diagnoses of bone marrow carcinomatosis of unknown primary origin and CR‐TMA were made. Then, fresh frozen plasma was administered for the DIC, and lactated Ringer's solution was administered for dehydration. Plasma exchange (PEX) was not performed, as the patient's PLASMIC score^4^ (two points; levels of hemolysis and MCV) indicated that she was low risk and would not benefit from it. Her laboratory values on the third hospital day were as follows: hemoglobin concentration, 7.1 g/dL; platelets, 177 × 10^9^/L; PT, 14.0 s; APTT, 28.8 s; fibrinogen, 130 mg/dL; FDP, 114.8 μg/mL; d‐dimer, 21.0 μg/mL; LDH, 2488 U/L. Lower intestinal endoscopy and fluorodeoxyglucose positron emission tomography (PET) were scheduled to search for the primary carcinoma; however, the patient's renal failure rapidly progressed before the primary tumor could be identified, and she died on the third hospital day. Consent for an autopsy could not be obtained from the patient's family.

## 3 DISCUSSION

Microangiopathic hemolytic anemia (MAHA) is a condition in which erythrocytes are mechanically restricted when passing through small blood vessels, resulting in erythrocyte degradation and hemolysis. MAHA may occur in isolation due to a direct effect on red blood cells, such as trauma caused by mechanical heart valves, infections (e.g., malaria) or march hemoglobinuria, but it more commonly occurs as a component of TMA. CR‐TMA is a rare type of Coombs‐negative hemolytic anemia, involving fragmented red blood cells, and is usually associated with thrombocytopenia and/or multiorgan microvascular thrombosis. Although the fact that thrombocytopenia was not seen in the present case seemed to be atypical, it was previously reported that a small number of patients with CR‐TMA may have exhibit a platelet count of ≥100 × 10^9^/L at the initial presentation.^5^ Regarding the pathogenesis of CR‐TMA, it has been suggested that it may be caused by fibrinoid necrosis of the bone marrow due to bone marrow carcinomatosis and tumor cell emboli affecting the arteries, arterioles, and/or capillaries.^6^ Red‐cell fragmentation and direct platelet destruction in the small blood vessels of cancerous tissue may also cause CR‐TMA in patients with solid cancer.^1^ In addition, in cases of adenocarcinoma high‐mucin expression may play a role in the pathogenesis of CR‐TMA due to mucin‐induced erythrocytopathy.^2^, ^3^ However, the exact mechanism underlying CR‐TMA has not been determined. In a clinical study of 154 cases of solid tumors accompanied by CR‐TMA, it was reported that adenocarcinoma was the most common histological type.^1^ As for the primary sites, gastric cancer, breast cancer, prostate cancer, and lung cancer were the most common sites (in that order), followed by cancer of unknown primary origin, as was found in our case.^1^ Although its frequency has not been calculated based on an exact number of cases, the incidence of CR‐TMA is extremely low. For example, in a study of breast carcinoma, the second most common cause of CR‐TMA, it was estimated to be less than 0.1%.^2^

TMA has various etiologies, which share similar laboratory and clinical characteristics. Determining the underlying etiology of TMA is critical for ensuring that appropriate treatment can be administered. TTP is a life‐threatening condition arising from primary hemostatic abnormalities. Under physiological conditions, von Willebrand factor (VWF) mediates platelet binding to subendothelial collagen at sites of vascular injury. During this process, activated endothelial cells release VWF in multimeric forms, which are then cleaved by the ADAMTS13 metalloproteinase. In TTP patients, a deficiency or impairment in ADAMTS13 leads to the accumulation of ultra‐large VWF multimers, causing the formation of microvascular platelet–VWF plugs, which may cause red blood cell shearing. ADAMTS13 activity levels below 10% are diagnostic of TTP, whereas level exceeding 20% indicate that this diagnosis is unlikely.^7^ The PLASMIC score, which incorporates seven variables, is a useful gage of the pretest probability that a patient has TTP.^4^ Due to the high‐morbidity rate associated with delays in the treatment of TTP, patients with TMA and a high PLASMIC score are often treated empirically for TTP until the results of ADAMTS13 testing become available.^7^ In the present case, the patient's low‐risk PLASMIC score made a diagnosis of TTP unlikely; hence, we avoided PEX, which has no benefit for CR‐TMA. It has been reported that the PLASMIC score is useful for differential diagnosis and avoiding unnecessary PEX, such as in the present case.^8^

DIC is a form of MAHA that arises from abnormal activation of the coagulation cascade, leading to microvascular thrombosis, the consumption of endogenous anticoagulants, excessive generation of fibrin, and the development of fibrinolytic derangements.^9^ Certain cancers are more likely to provoke DIC, particularly adenocarcinomas of the gastrointestinal tract (especially the signet‐ring cell type), pancreas, breast, prostate, or lung, and mucinous tumors may secrete enzymes that can activate factor X.^10^ In addition, DIC is strongly associated with CR‐TMA.^1^, ^5^ For example, it was suggested that the generation of small‐vessel thrombi in CR‐TMA may be increased by simultaneous DIC.^5^ The laboratory findings of our case were indicative of overt DIC, which may have been driven by the cancer itself. As another possibility, several drugs have been reported to cause TMA.^11^ Drug‐induced TMA may be immunologically mediated by antibodies with specificity against endothelial or other inflammatory cells, or it may arise as a result of the toxic effects of a drug on endothelial cells. However, our patient was not taking any medications that are associated with TMA, which made drug‐induced TMA unlikely.

Concerning diagnosis, CR‐TMA is generally accompanied by multiple bone or bone marrow metastases. Hence, bone marrow investigations are useful as a primary diagnostic method for identifying the underlying occult carcinoma.^6^, ^12^ In the present case, the presence of an underlying causative tumor was strongly suspected because of the patient's high carcinoembryonic antigen and neuron‐specific enolase levels, and a diagnosis of epithelial carcinoma was made after a bone marrow examination. Clusters of tumor cells were observed in a smear as well as in a clot section, leading to a diagnosis of bone marrow carcinomatosis and related TMA. It has been pointed out that clot sections are useful in that they allow additional examinations to be performed when metastatic tumors are found because they are not subjected to decalcification.^13^ Although the primary carcinoma could not be identified in the present case, it was possible to narrow down the likely origin of the primary tumor by subjecting the clot specimen to immunostaining. The morphology of the tumor cells led to a histopathological diagnosis of suspected signet‐ring cell carcinoma. It has been reported that adenocarcinoma is the most common histological type among the causative diseases associated with CR‐TMA.^1^ As for the reason why carcinoma of unknown primary origin exhibits a higher frequency in patients with CR‐TMA than in the general population, even if a primary tumor is so small that it cannot be detected due to disseminated tumor cell dormancy,^14^ the rapid dissemination of metastases to the bone and/or bone marrow may lead to the development of CR‐TMA. In addition, concerning diagnostic delays, it has been reported that it takes an average of 6 days to diagnose the primary lesion if CR‐TMA is diagnosed first.^15^ In the meantime, it is possible that the original disease will worsen and the patient will die before the primary tumor is identified; therefore, such patients must be diagnosed with carcinoma of unknown primary origin, as in the present case. In fact, in this case CT revealed no abnormal findings that were suggestive of a primary tumor, and lower gastrointestinal endoscopy and PET were planned; however, the patient's disease progressed rapidly, and she died before these examinations could be performed. However, immunohistochemical staining of the bone marrow clot section was negative for GATA3, which ruled out breast cancer.^16^ On the other hand, decreased CDX2 expression is associated with poor differentiation and MMR deficiency in colorectal cancer.^17^ In addition, it has been reported that elevated neuron‐specific enolase levels are seen in patients with advanced colorectal cancer.^18^ Thus, although the patient had to be diagnosed with cancer of unknown primary origin, as lower gastrointestinal endoscopy was not performed, and the results of MMR deficiency‐related staining were inconclusive, it was assumed that colorectal cancer was likely to be the primary disease.

As for therapeutic interventions, chemotherapy is the only effective treatment for CR‐TMA. There have been a few reported cases in which chemotherapy was effective against carcinoma of unknown primary origin accompanied by CR‐TMA.^1^, ^19^, ^20^ However, many patients have an extremely limited prognosis, with the condition exhibiting a mortality rate of nearly 50% within 1 month of diagnosis.^1^, ^10^ Hence, when a diagnosis of CR‐TMA is established it is necessary to make an urgent decision, whether it is best to treat the patient with appropriate chemotherapy or plan for palliative care.

## 4 CONCLUSION

We presented a case of CR‐TMA caused by bone marrow carcinomatosis due to carcinoma of unknown primary origin. In the present case, the PLASMIC score was useful for ruling out TTP, which requires emergency PEX. Although the primary tumor could not be identified, immunohistochemical staining and tumor marker data suggested that colorectal cancer was the primary disease. Although MMR‐related tests were inconclusive in the current case, it may be possible to strengthen a diagnosis of MMR‐deficient colorectal cancer in cases in which MMR‐deficiency is proven.

## AUTHOR CONTRIBUTIONS

**Masahiro Manabe:** Conceptualization (equal); data curation (equal); writing – original draft (equal); writing – review and editing (equal). **Naoyuki Inano:** Data curation (equal); writing – review and editing (equal). **Yuuji Hagiwara:** Data curation (equal); writing – review and editing (equal). **Nobuhiro Sogabe:** Data curation (equal); writing – review and editing (equal). **Satoru Nanno:** Data curation (equal); writing – review and editing (equal). **Takeshi Mazaki:** Data curation (equal); supervision (equal). **Ki‐Ryang Koh:** Conceptualization (equal); supervision (equal); writing – original draft (equal); writing – review and editing (equal).

## CONFLICT OF INTEREST STATEMENT

The authors have stated explicitly that there are no conflicts of interest in connection with this article.

## ETHICS STATEMENT

This study was conducted as per the Declaration of Helsinki.

## PATIENT CONSENT STATEMENT

Written informed consent was obtained from the patient for the publication of this study.

## Data Availability

None.
